# Ultrasound-guided vs. fluoro-guided axillary venous access for cardiac implantable electronic devices: a patient-based meta-analysis

**DOI:** 10.1093/europace/euae274

**Published:** 2024-10-29

**Authors:** Francesco Vitali, Marco Zuin, Paul Charles, Javier Jiménez-Díaz, Seth H Sheldon, Ana Paula Tagliari, Federico Migliore, Michele Malagù, Mathieu Montoy, Felipe Higuera Sobrino, Alex M Courtney, Adriano Nunes Kochi, Samir Fareh, Matteo Bertini

**Affiliations:** Cardiology Department, Sant’Anna University Hospital, University of Ferrara, Via Aldo Moro 8, 44124 Cona, Ferrara, Italy; Cardiology Department, Sant’Anna University Hospital, University of Ferrara, Via Aldo Moro 8, 44124 Cona, Ferrara, Italy; Hôpital de la Croix Rousse, Hospices Civils de Lyon, Lyon, France; Arrhythmia Unit, Cardiology Department, Hospital General Universitario of Ciudad Real, Ciudad Real, Spain; Department of Cardiovascular Medicine, The University of Kansas Medical Center, Kansas City, KS, USA; Program in Cardiology and Cardiovascular Sciences, School of Medicine, Universidade Federal do Rio Grande do Sul, Porto Alegre, Brazil; Department of Cardiovascular Surgery, Hospital Mãe de Deus, Porto Alegre, Brazil; Department of Cardiac, Thoracic and Vascular Sciences and Public Health, University of Padova, Padua, Italy; Cardiology Department, Sant’Anna University Hospital, University of Ferrara, Via Aldo Moro 8, 44124 Cona, Ferrara, Italy; Hôpital de la Croix Rousse, Hospices Civils de Lyon, Lyon, France; Arrhythmia Unit, Cardiology Department, Hospital General Universitario of Ciudad Real, Ciudad Real, Spain; Hôpital de la Croix Rousse, Hospices Civils de Lyon, Lyon, France; Department of Cardiovascular Surgery, Hospital Mãe de Deus, Porto Alegre, Brazil; Hôpital de la Croix Rousse, Hospices Civils de Lyon, Lyon, France; Cardiology Department, Sant’Anna University Hospital, University of Ferrara, Via Aldo Moro 8, 44124 Cona, Ferrara, Italy

**Keywords:** Axillary venous access, Ultrasound, Venous access, Pacemaker, ICD

## Abstract

**Aims:**

The use of ultrasound (US)-guided venous puncture for cardiac pacing/defibrillation lead placement may minimize the risk of periprocedural complications and radiation exposure. However, none of the published studies have been sufficiently powered to recommend this approach as the standard of care. We compare the safety and efficacy of ultrasound-guided axillary venous puncture (US-AVP) vs. fluoroscopy-guided access for cardiac implantable electronic devices (CIEDs) by performing an individual patient data meta-analysis based on previously published studies.

**Methods and results:**

We conducted a thorough literature search encompassing longitudinal investigations (five randomized and one prospective studies) reporting data on X-ray-guided and US-AVP for CIED procedures. The primary endpoint was to compare the safety of the two techniques. Secondary endpoints included the success rate of each technique, the necessity of switching to alternative methods, the time needed to obtain venous access, X-ray exposure, and the occurrence of periprocedural complications. Six longitudinal eligible studies were identified including 700 patients (mean age 74.9 ± 12.1 years, 68.4% males). The two approaches for venous cannulation showed a similar success rate. The use of an X-ray-guided approach significantly increased the risk of inadvertent arterial punctures (OR: 2.15, 95% CI: 2.10–2.21, *P* = 0.003), after adjustment for potential confounders. Conversely, a US-AVP approach reduces time to vascular access, radiation exposure, and the number of attempts to vascular access.

**Conclusion:**

The US-AVP enhances safety by reducing radiation exposure and time to vascular access while maintaining a low rate of major complications compared to the X-ray-guided approach.

**Clinical trial registration:**

PROSPERO identifier: CRD42024539623.

## Graphical Abstract

**Graphical Abstract:**
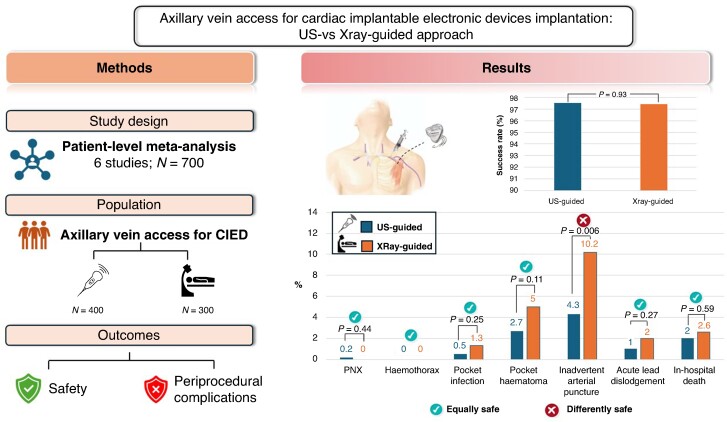

**What’s new?**

- Ultrasound axillary vein puncture enhances safety by reducing radiation exposure and time to vascular access.
- Ultrasound axillary vein puncture is associated with a low rate of major complications compared to the X-ray-guided approach.
- Ultrasound axillary vein puncture should be regarded as the optimal choice for venous access during cardiac implantable electronic device implantation.

## Introduction

Venous access is a crucial component of cardiac implantable electronic device (CIED) implantation and often presents significant challenges. Current international guidelines emphasize the necessity of proficiency in axillary vein puncture (AVP) to ensure successful CIED implantation.^1^ In previous randomized controlled trials (RCTs) and observational studies, both cephalic vein cutdown (CVC) and AVP have shown a superior safety profile for both acute and chronic complications compared to subclavian vein puncture (SVP), exhibiting lower rates of acute pneumothorax, haemothorax, and lead fracture during follow-up.^2–4^ From an interventional perspective, CVC may take longer than SVP and AVP, and its use may be restricted by the smaller calibre of the cephalic vein or challenges in accessing it. Conversely, AVP also appears to be the safest and fastest modality of vascular access for CIED implantation.^4^ However, in daily clinical practice, AVP is frequently performed under fluoroscopic guidance, relying solely on bony landmarks,^5^ especially when venograms are not performed.^6^ This reliance on fluoroscopy can present challenges, particularly if inadvertent puncture of the axillary artery occurs, potentially leading to vein compression due to periarterial haematoma.

Previous clinical investigations have shown that an ultrasound-guided AVP (US-AVP) during CIED implantations is safe and effective, minimizing the risk of major periprocedural complications.^7^ However, these studies were not sufficiently powered to provide solid evidence recommending the US-AVP as the standard of care. Given the paucity of evidence and to partially address this knowledge gap, we conducted an individual patient-level meta-analysis to compare the efficacy and safety of a US-AVP vs. the X-ray-guided approach in patients undergoing CIED implantation.

## Methods

### Study design, search strategy, and selection criteria

A systematic review of published research providing data on the safety and efficacy between a US-AVP and an X-ray-guided method for CIED procedures was conducted in accordance with the Preferred Reporting Items for Systematic Review and Meta-Analyses of Individual Participant Data (PRISMA-IPD).^8^ The protocol for this systematic review and individual patient data meta-analysis (IPDMA) was prospectively registered with PROSPERO (identifier: CRD42024539623). Each study included into the present investigation was approved by local ethics committees, and all patients provided written informed consent.

All relevant publications were identified by searching MEDLINE and Scopus from inception up to March 2024. The full search strategy is presented in the Supplementary material online, *Table S1*. Moreover, we searched the bibliographies of the target studies for additional references. The study selection was independently conducted by two authors (F.V. and M.Z.) in a blinded fashion. Any discrepancies in study selection were resolved by consulting a third author (M.B.) and resolved by collegial discussion. Access to anonymized patient-level data was granted from the investigators of the studies included.

Inclusion criteria were: (i) randomized trials or prospective studies, (ii) written in English, (iii) comparing US-guided vascular access to fluoroscopic-AVP, performed irrespectively of venography, or (iv) reporting the occurrence of periprocedural complications and in-hospital mortality. Patients who underwent CVC or SVP were not included in the analysis.

### Risk of bias assessment

Two investigators (Mi.M. and M.Z.) independently assessed the risk of bias using the revised Cochrane risk-of-bias tool (RoB 2)^9^ and the Newcastle-Ottawa quality assessment scale (NOS)^10^ for RCTs and prospective studies, respectively. Disagreements, if any, were solved by discussion and, if unsolved, by consulting a third investigator (F.V.).

### Study population

A patient-level database was created by merging single studies databases provided by the studies’ coordinators. Then, individual patient data were centrally homogenized and merged in a pooled electronic database that was housed at the University of Ferrara, Department of Cardiology. Data were checked for integrity and completeness, and the clean data were subsequently analysed. Variables were identified, measurements verified, and comparisons with individual reports were made. Discrepancies with the published data, if any, were resolved by contacting the principal investigators. The following data were collected: demographics, body mass index (BMI) in kg/m^2^, previous history of myocardial infarction, atrial fibrillation, diabetes, chronic kidney disease (CKD), left ventricular ejection fraction (LVEF), and use of antiplatelets or anticoagulant drugs. Strict protocols were followed in all the revised investigations to ensure that, for patients receiving a US-guided approach, the probe remained sterile in the operating field, thereby minimizing the risk of infection. Moreover, we obtained data regarding the type of device, procedural data, and in-hospital outcomes/complications (see Supplementary material online, *Tables S2* and *S3*). The different inclusion and exclusion criteria used in revised manuscripts are presented in Supplementary material online, *Table S4*.

### Study outcomes

The primary endpoint of the study was to compare the safety in terms of acute complications of US-guided vs. X-ray-guided approaches for axillary venous access cannulation during PM or ICD implantation. Indeed, as stated in the introduction, none of the published studies comparing US vs. X-ray-guided approaches for CIED implantation possess the adequate statistical power to definitively recommend one technique over the other for venous cannulation. Secondary endpoints included the success rates of the US- and X-ray-guided approaches, the need to switch to alternative techniques, the time required to obtain venous access, X-ray exposure, the occurrence of periprocedural complications, and in-hospital mortality. Furthermore, given that the existing data yielded varied and inconsistent results concerning the impact of puncture sites for AVP, we conducted a sub-analysis to investigate the implications of employing an ‘inside’ vs. ‘outside’ pocket strategy, utilizing a large real-world cohort.^7^

### Statistical analysis

We conducted a descriptive analysis of baseline clinical characteristics and outcomes, stratifying the population into two groups based on whether a US-guided or X-ray-guided approach was used for the cannulation of the AV during CIED procedures. Distribution of continuous variables was tested for normality with the Shapiro–Wilk test. Continuous variables were reported as mean ± standard deviation or median (25th–75th percentile) and analysed with a Student’s *t*-test. Categorical variables were reported as percentage and compared with χ^2^ or Fisher’s exact test. To investigate the relationship between the risk of inadvertent arterial puncture among patients receiving a US- or an X-ray-guided approach, a multivariate regression analysis with the corresponding 95% confidence intervals (CIs) was performed. Specifically, a *P*-value of <0.10 was pre-defined as the cut-off for inclusion of the univariate parameters into the multivariate logistic regression model. A two-sided *P*-value of <0.05 was considered statistically significant. All analyses were performed in SPSS (version 26.0) and R (version 4.3.2) (R Foundation for Statistical Computing; www.Rproject.org), by using the lme4 package.

## Results

### Included studies

**Figure 1:**
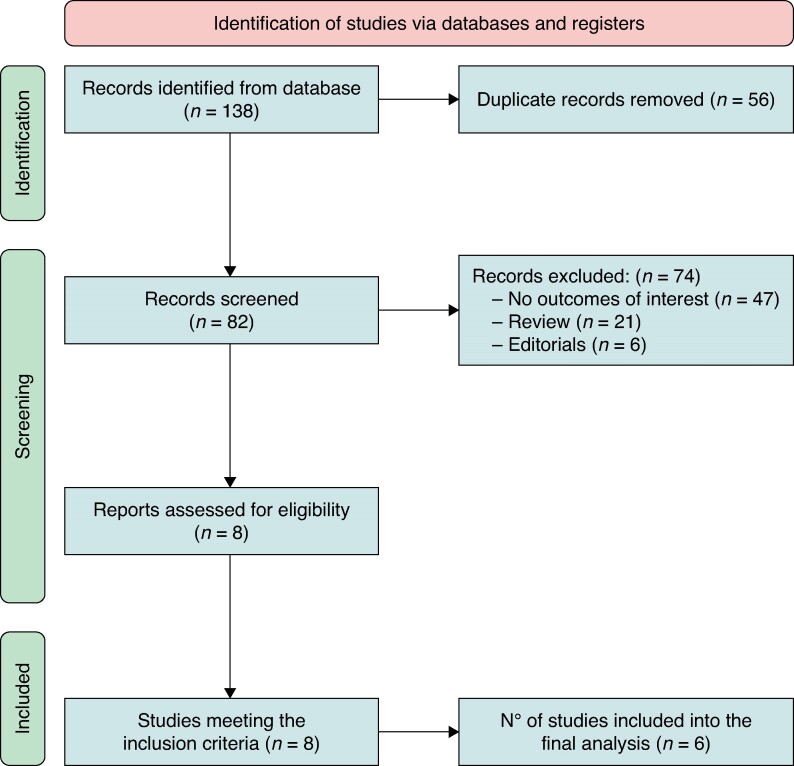
PRISMA flowchart.

**Figure 2:**
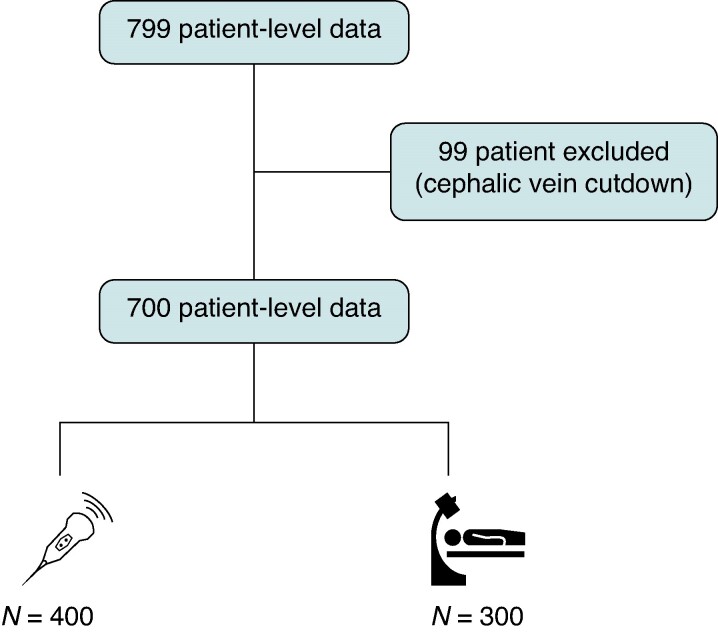
Study flowchart.

Among 82 studies identified by using our research strategy, eight fulfilled the inclusion criteria (*Figure 1*). Of these, six datasets were obtained with individual patient data, while two studies were excluded because the authors did not reply to the data^7,11–15^ (*Figure 1*). Overall, 700 patients (mean age 74.9 ± 12.1 years, 68.4% males) (*Figure 2*) were included into the final analysis (*Tables 1* and *2*). Characteristics of included studies, and risk of bias assessment according to the NOS and RoB 2, are shown in Supplementary material online, *Tables S5* and *S6*.

**Table 1:** General characteristics of the patients enrolled in the reviewed investigations, treated using a ultrasound-guided or an X-ray fluoroscopic-guided approach for axillary vein puncture

	Vitali *et al*.^11^	Courtney *et al*.^13^	Jimenez *et al*.^15^	Charles *et al*.^7^	Migliore *et al*.^12^	Tagliari *et al*.^14^
Study settings and demographics						
Country	Italy	USA	Spain	France	Italy	Brazil
Study design	Randomized	Randomized	Randomized	Randomized	Prospective	Randomized
No. of patients	270	100	120	101	95	44
Mean age (years)	78.5	67.9	74.9	77.0	78	64.6
Males, *n* (%)	159 (58.9)	68 (68.0)	73 (60.8)	70 (69.3)	58 (61.0)	26 (59.1)
Comorbidities and CV risk factors						
HT, *n* (%)	227 (84.1)	79 (79.0)	101 (84.2)	60 (59.4)	NR	28 (63.6)
Previous MI, *n* (%)	80 (30)	64 (64.0)	26 (21.7)	27 (26.7)	NR	8 (18.2)
AF, *n* (%)	95 (35.2)	38 (38.0)	43 (35.8)	23 (22.8)	24 (25.0)	8 (18.2)
DM, *n* (%)	82 (30.4)	31 (31.0)	42 (35.0)	29 (28.7)	NR	18 (40.9)
HF, *n* (%)	88 (32.6)	59 (59.0)	29 (24.2)	23 (22.8)	NR	13 (29.5)
Mean LVEF, (%)	53.8	47.5	56.2	60.1	NR	51.2
CKD, *n* (5)	158 (58.5)	40 (40.0)	19 (15.8)	41 (40.6)	NR	6 (13.6)
Treatments						
Antiplatelet, *n* (%)	229 (84.8)	64 (64.0)	30 (25.30)	34 (33.7)	34 (36.0)	4 (9.1)
Anticoagulants, *n* (%)	95 (35.2)	38 (38.0)	43 (35.8)	35 (34.7)	24 (25.0)	10 (22.7)

HT, arterial hypertension; DM, diabetes mellitus; HF, heart failure; LVEF, left ventricular ejection fraction; CKD, chronic kidney disease.

**Table 2:** Demographics and baseline clinical characteristics

	Entire cohort	US-AVP	X-ray-AVP	*P*
	*n* = 700	*n* = 400	*n* = 300	
Demographics				
Age (years)	74.9 ± 12.1	74.1 ± 13.2	75.9 ± 11.9	0.07
Males, *n* (%)	454 (64.8)	248 (62.0)	206 (68.6)	0.15
Body mass index (kg/m^2^)	29.2 ± 5.0	26.0 ± 2.2	29.4 ± 5.0	0.10
Cardiovascular comorbidities				
Hypertension, *n* (%) [*n* = 635]^a^	495 (77.9)^a^	262 (56.7)^a^	233 (82.0)^a^	0.02
Previous MI, *n* (%) [*n* = 635]^a^	352 (55.4)^a^	199 (56.7)^a^	153 (53.9)^a^	0.48
History of HF, *n* (%) [*n* = 635]^a^	212 (33.3)^a^	123 (35.1)	89 (31.3)	0.30
AF, *n* (%)	231 (36.3)^a^	126 (31.5)	105 (35.0)	0.33
Diabetes, *n* (%) [*n* = 635]^a^	202 (31.8)^a^	111 (31.6)^a^	91 (32.0)^a^	0.91
CKD, *n* (%) [*n* = 635]^a^	264 (41.5)^a^	160 (45.6)^a^	104 (36.6)^a^	0.02
LVEF, (%) [*n* = 635]^a^	54.1 ± 12.5	53.8 ± 13.4	54.5 ± 11.4	0.51
Antithrombotic treatments				
Antiplatelets, *n* (%)	395 (56.4)	217 (54.2)	178 (59.3)	0.18
Anticoagulants, *n* (%)	245 (35.0)	140 (35.0)	105 (35.0)	0.99
Device type				
PM, *n* (%)	534 (76.2)	297 (74.2)	237 (79.0)	0.14
ICD, *n* (%)	166 (23.7)	90 (22.5)	76 (25.3)	0.39
Active fixation leads, *n* (%)	578 (82.5)	296 (74.0)	282 (94.0)	<0.001

AF, atrial fibrillation; CKD, chronic kidney disease; LVEF, left ventricular ejection fraction; MI, myocardial infarction.

^a^Based on 635 patients (351 in the US-AVP and 284 in the X-ray-AVP group, respectively).

### Demographic and baseline clinical features: ultrasound-guided axillary venous puncture vs. X-ray-axillary vein puncture

The demographics and baseline clinical characteristics of patients treated using a US-guided or X-ray-guided approach are presented in *Table 1*. The two groups did not differ in age, sex, or BMI. Patients receiving AVP cannulation using the X-ray-guided method had higher prevalence of arterial hypertension compared to those who underwent the US-guided approach (82.0% vs. 74.6%, *P* = 0.02). Conversely, a history of CKD was more common in patients treated with the US-guided approach (45.6% vs. 36.6%, *P* = 0.02). No differences were observed regarding the use of antiplatelet or anticoagulant drugs between the two groups.

#### Procedural findings

**Figure 3:**
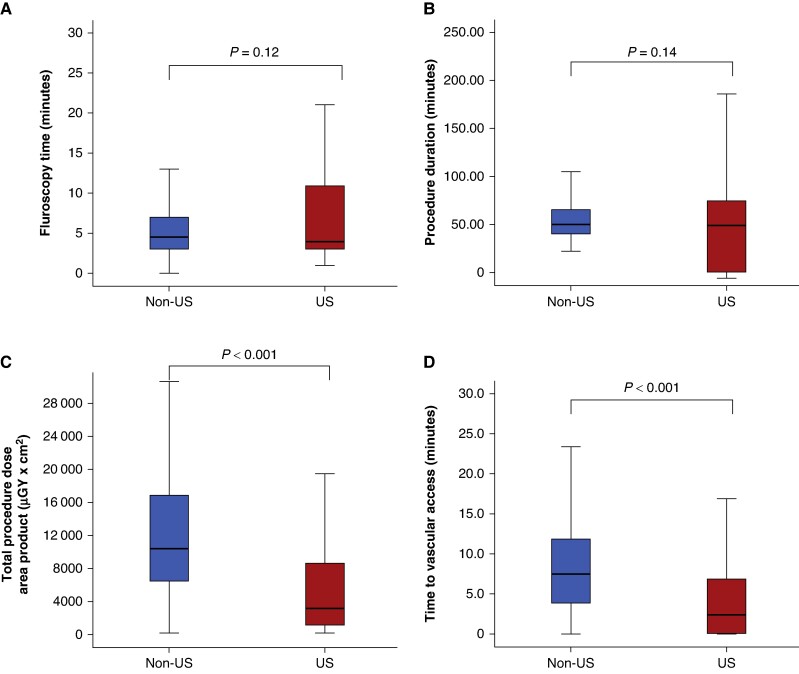
Differences for secondary endpoints between X-ray-AVP (blue) and US-AVP (red). (*A*) Differences in total fluoroscopy time. (*B*) Differences in total procedure duration. (*C*) Differences in total procedure dose area product. (*D*) Differences in time to vascular access.

No differences in the success rate of AVP were observed between the two groups (US-AVP 97.5% vs. X-ray-AVP 97.4%, *P* = 0.93). Specifically, patients treated with a US-guided approach more frequently received a puncture inside the pocket (82.9% vs. 38.0%, *P* < 0.001), whereas those treated with an X-ray-guided approach more often received a puncture prior to incision through the skin (61.9% vs. 37.6%, *P* < 0.001). Moreover, patients treated with a US-guided approach had lower access X-ray dose area products and needed fewer attempts to cannulate the AV. Conversely, no differences were observed regarding the cross-over to a different vascular access/imaging modality (*Table 3*). Moreover, patients receiving a US-guided approach exhibited lower total procedure dose area product (*P* < 0.001) and faster vascular access acquisition times (*P* < 0.001), with comparable total fluoroscopy time and overall procedure duration across both study groups (*Figure 3*).

**Table 3:** Procedural data

	Entire cohort	US-AVP	X-ray-AVP	*P*
	*n* = 700	*n* = 400	*n* = 300	
Puncture inside the pocket, *n* (%) [*n* = 459]^a^	327 (71.2)	219 (82.9)	108 (38.0)	<0.001
Puncture prior to incision through the skin, *n* (%) [*n* = 459]^a^	308 (67.1)	132 (37.6)	176 (61.9)	<0.001
No. of axially puncture, *n*	2.3 ± 0.5	1.8 ± 0.3	2.8 ± 0.5	<0.001
Vascular access dose area product, µGy × cm^2^ [IQR]	573.5 [0–3568.2]	0 [0–1628]	10 344.5 [6363–16 870]	<0.001
No. of attempts for vascular access	1.8 ± 0.3	1.3 ± 0.2	2.4 ± 0.3	<0.001
Cross-over to different vascular access/imaging modality [*n* = 635]^b^	94 (13.4)	54 (15.4)	40 (14.1)	0.64
Axillary vein puncture failure	18 (2.5)	10 (2.5)	8 (2.6)	0.93

IQR, interquartile range.

^a^Based on 459 patients (351 in the US-AVP and 108 in the X-ray-AVP group, respectively).

^b^Based on patients (351 in the US-AVP and 284 in the X-ray-AVP group, respectively).

#### Periprocedural complications

Regarding the occurrence of periprocedural complications, the two groups had a similar incidence of pneumothorax (*P* = 0.44), pocket infection (*P* = 0.25), haematoma (*P* = 0.11), and acute lead dislodgement (*P* = 0.27). However, a higher incidence of inadvertent arterial puncture was observed in patients receiving an X-ray-guided approach (*P* = 0.006) (*Table 4* and Supplementary material online, *Table S7*). Multivariate regression analysis confirmed that AVP cannulation using an X-ray-guided approach was associated with a higher risk of inadvertent arterial puncture (OR: 2.15, 95% CI: 2.10–2.21, *P* = 0.003), independently by the use of antiplatelets or anticoagulants (OR: 2.36, 95% CI: 1.08–3.21, *P* = 0.01), puncture strategies as inside the pocket or prior to incision through the skin (OR: 1.74, 95% CI: 1.68–1.83, *P* < 0.001), and history of CKD (OR: 1.53, 95% CI: 1.38–1.72, *P* = 0.01) (*Table 5*). No differences in the in-hospital mortality rate were observed between the two groups (*P* = 0.59).

**Table 4:** Occurrence of periprocedural complications and in-hospital death

	Entire cohort	US-AVP	X-ray-AVP	*P*
	*n* = 700	*n* = 400	*n* = 300	
PNX, *n* (%)	1 (0.1)	1 (0.2)	0	0.44
Haemothorax, *n* (%)	0	0	0	–
Pocket infection, *n* (%)	6 (0.8)	2 (0.5)	4 (1.3)	0.25
Pocket haematoma, *n* (%)	26 (3.7)	11 (2.7)	15 (5.0)	0.11
Inadvertent arterial puncture, *n* (%)	39 (7.0)	13 (4.3)	26 (10.2)	0.006
Acute lead dislodgment, *n* (%)	10 (1.4)	4 (1.0)	6 (2.0)	0.27
In-hospital death, *n* (%)	16 (2.2)	8 (2.0)	8 (2.6)	0.59

PNX, pneumothorax.

**Table 5:** Univariate and multivariate analyses for the risk of inadvertent arterial puncture

	Univariate		Multivariate	
	OR [95% CI]	*P*	OR [95% CI]	*P*
X-ray-guided vs. US-guided puncture strategy	2.34 [1.26–4.28]	0.006	2.15 [2.10–2.21]	0.003
Age ≥ 65	0.97 [0.81–1.14]	0.78	–	
BMI ≥ 30	0.82 [0.77–1.15]	0.83	–	
Antiplatelets/anticoagulants (no vs. yes)	2.41 [1.12–4.36]	0.02	2.36 [1.08–3.21]	0.01
Active fixation leads (no vs. yes)	0.49 [0.22–1.49]	0.84	–	
Sites of puncture (inside the pocket vs. prior to incision through the skin)	1.84 [1.70–1.92]	<0.001	1.74 [1.68–1.83]	<0.001
CKD	1.97 [1.83–2.15]	0.002	1.53 [1.38–1.72]	0.01

BMI, body mass index; CKD, chronic kidney disease; CI, confidence interval; US, ultrasound.

#### Puncture inside the pocket or prior to incision through the skin

**Figure 4:**
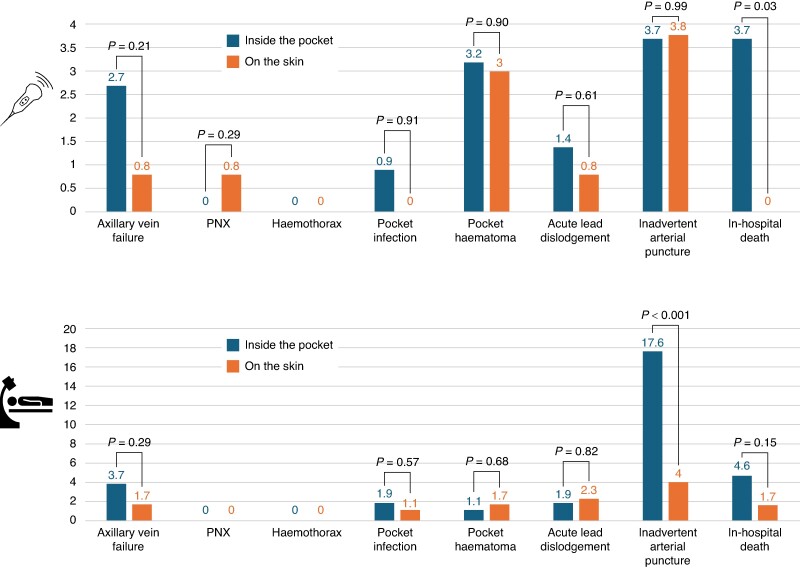
Comparison of periprocedural complications and in-hospital mortality between US-AVP (upper part) and X-ray-AVP (lower part). Highlighted differences between two types of punctures for both study groups: inside the pocket (blue bars) and directly on the skin (orange bars).

A subgroup analysis was performed to evaluate potential differences in periprocedural complications among patients treated with either a US-guided or X-ray-guided approach, considering the type of AVP puncture: inside the pocket or prior to incision through the skin. In the US-guided group, no significant differences were observed in periprocedural complications. However, a higher mortality rate was noted among patients receiving the puncture inside the pocket, who were older (77.8 ± 10.8 vs. 71.9 ± 13.8, *P* < 0.001), and had a higher prevalence of arterial hypertension (67.0 vs. 43.2, *P* < 0.001) and CKD (52.9% VS 29.5%, *P* < 0.001), and more frequently received antiplatelets or oral anticoagulants (68.5% vs. 44.5%, *P* < 0.001) compared to those who received the puncture on the skin. Conversely, in the X-ray-guided group, the occurrence of inadvertent arterial puncture was the only significant periprocedural complication, occurring more frequently in patients receiving a puncture inside the pocket (*P* < 0.001) (*Figure 4*).

## Discussion

This patient-level meta-analysis suggests the following:

1. Both US-guided and X-ray-guided approaches demonstrated comparable success rates for AV cannulation. However, the US-guided approach reduced both total radiation exposure and vascular access time.
2. Both techniques exhibited similarly low rates of acute periprocedural complications. Ultrasound-guided axillary venous puncture cannulation, by enabling direct visualization of the needle tip and surrounding structures, significantly mitigated the risk of inadvertent arterial puncture.
3. In the X-ray-guided group, inadvertent arterial puncture was more frequent among patients who underwent puncture inside the pocket.

Presently, AVP is primarily performed under fluoroscopic guidance, a method that has demonstrated superior outcomes in terms of acute safety and efficacy when compared to SVP and CVC.^4,5^ Despite the significant interest in AVP, the technique for its execution has remained largely unchanged since its initial description and standardization.^16^ While other methods such as venograms^6^ and caudal fluoroscopy views^17^ have shown effectiveness in specific cases, the persistent challenge lies in the inability to directly visualize the AV and surrounding structures like the artery and pleural line. These limitations restrict the widespread adoption of this vascular access approach, especially in overweight/underweight patients and those with a high clavicular take-off.

The current patient-level meta-analysis corroborates previous findings^7,11^ indicating no significant difference in the occurrence of major complications related to vascular access between US- and fluoroscopic-guided strategies. The observed mortality rate has likely been influenced by several factors, including the age of the enrolled patients and their burden of pre-existing comorbidities, which may have contributed to the exacerbation of chronic cardiovascular and non-cardiovascular conditions, thereby affecting the mortality rate. The observed differences in total procedure dose area product between the groups may be attributed to variations in X-ray power and strength utilized across different studies, particularly since three of the studies exclusively included patients undergoing X-ray-guided procedures.

Furthermore, present findings indicate that the primary difference between ultrasound-guided and fluoroscopy-guided AVP in terms of procedural complications was a lower rate of inadvertent axillary arterial punctures (4% vs. 10%; *P* < 0.001). This observation was consistent in multivariable analysis, where fluoroscopy-guided AVP significantly increased the risk of inadvertent arterial punctures (OR: 2.15, 95% CI: 2.10–2.21, *P* = 0.003) after adjusting for potential confounders. While AVP generally presents fewer complications compared to CVC and SVP, inadvertent axillary artery puncture remains a common intraprocedural issue that can prolong procedural time and elevate the risk of major complications like significant bleeding and infections.^7^ Unexpectedly, we did not observe any difference in the rate of pneumothorax (PNX) between the two groups. This may be attributed to the fact that all the reviewed investigations were performed in highly specialized centres by experienced operators; these settings and operators could have distorted our results compared to what is commonly observed in daily clinical practice. Moreover, the lower incidence of PNX may be attributed to the fact that all the reviewed investigations were conducted in experienced centres. Indeed, previous literature has reported an incidence of pneumothorax ranging between 1% and 2% in daily clinical practice.^7,18,19^ Probably, in this context, the use of a US approach may be more advantageous for less experienced operators, potentially reducing the rate of intraoperative complications. The use of a US-guided approach may be more advantageous for less experienced operators, potentially reducing the rate of intraoperative complications. Ultrasound provides direct visualization of the AV and surrounding structures, along with real-time needle tip monitoring, thereby reducing the likelihood of inadvertent arterial puncture. Our findings support that US-AVP is a more practical and safer method than X-ray-AVP alone. Additionally, employing ultrasound for venous access diminishes radiation exposure for both patients and operators.^20^

Our findings highlighted that vascular access was performed within the pocket in 83% of cases using US, while only 38% of patients with X-ray access had the same approach. Among these groups, we observed a significant difference in the incidence of inadvertent arterial puncture. In this regard, we cannot exclude the possibility that the results may have been influenced by a type II statistical error or some elements of operator bias. Therefore, our results cannot definitively affirm the superiority of one technique compared to the others but may provide an insightful overview on the different impact of a US- or X-guided strategy, for AVP, in the real-world. However, presented results seem to suggest that if the puncture is performed after the skin incision, it is better to use US guidance to reduce the risk of inadvertent arterial puncture.

There are two different modalities of AVP both with the use of US and under fluoroscopic guidance: puncturing prior to incision through the skin or puncturing inside the skin incision before creating the pocket for the device. Our results clarified important issues regarding the differences between these approaches. In the US-guided group, no significant differences were observed in periprocedural complications, on the other hand, in the X-ray-guided group, the occurrence of inadvertent arterial puncture occurred more frequently in patients receiving a puncture inside the pocket. Finally, US-AVP achieves a similar success rate compared to X-ray-AVP with fewer minor complications also reducing X-ray exposure for both operators and patients as recommended by international consensus papers^1^ making venous access easier and faster.

Ultrasound provides direct visualization of the AV and surrounding structures, along with real-time needle tip monitoring, thereby reducing the likelihood of inadvertent arterial puncture. It should be noted that several cases of inadvertent arterial puncture are self-limiting and do not result in significant serious clinical consequences; however, they can increase vascular access time, prolong the procedure, and make vascular access more difficult.^11^ However, the routine use of a US-AVP warrants consideration, as they can significantly impact its implementation in clinical practice. Key challenges include the need for specialized training to ensure that operators are proficient in the technique, which can involve a steep learning curve. Additionally, there may be an initial increase in procedural times as operators become familiar with the ultrasound equipment and technique. These factors could pose barriers to widespread adoption, particularly in settings where operators have limited experience with US guidance. Addressing these challenges through targeted training programmes and institutional support may be essential to facilitate the transition to US-AVP and maximize its potential benefits in improving patient safety and outcomes.

## Strengths and limitations

This is the first IPDMA comparing the safety and efficacy of US-AVP vs. a fluoroscopy-guided access for CIED. Published longitudinal investigations on the issue reflected the contemporary interventional management and the heterogeneous patient population encountered in clinical practice. Present analysis, for the first time in medical literature, provides a synthesis of current available evidence using different source of data, increasing statistical power and offering a more comprehensive perspective on the safety and efficacy of US-AVP vs. the use of a fluoroscopy-guided access for CIED. However, our study has also several limitations.

First, data collected through systematic reviews are inherently susceptible to selection and publication bias. We endeavoured to mitigate this by implementing various preventive measures for data collection, including contacting individual study authors and employing snowballing techniques. The limited number of studies included, as well as the relatively small sample size and the number of events, represents other important limitations. However, in the present study, we included almost all the published datasets on this issue. We endeavoured to mitigate this by implementing various preventive measures for data collection, including contacting individual study authors and employing snowballing techniques. Secondly, our analyses were stratified based on only two variables, which may be insufficient for identifying distinct groups of individuals. However, the selected phenotypes were characterized by substantial clinical uncertainty. Thirdly, while most endpoints were consistent across studies, an independent event adjudication committee evaluated their occurrence in only a minority of cases. Additionally, although we did not exclude any specific participant group, certain types of patients may have been underrepresented or excluded, cautioning against generalizing our findings to suggest equal treatment efficacy across all patient groups. Moreover, the inclusion of only a single population arm in our meta-analysis, particularly given that three of the six studies compared CVC to X-ray-guided axillary vein puncture—while excluding patients who underwent X-ray and ultrasound-guided axillary vein access—may have introduced biases that could affect the overall validity of our findings. The effectiveness of ultrasound and X-ray-guided techniques for vascular access during CIED implantation is influenced by various factors, including equipment availability, operator experience, and procedural variations, all of which may have partially biased our results. Moreover, we cannot exclude the possibility that the short-term follow-up may have missed some complications, such as infections, which can develop over a longer period. The absence of data regarding the use of specific materials, as well as micro-puncture needles, further limited our ability to perform dedicated sub-analyses. In addition, we were unable to explore the potential competing risk of death, which might have partially influenced our results. Finally, our exploration of short-term outcomes precludes definitive conclusions about long-term outcomes; thus, further studies are warranted to validate our findings over an extended period.

## Conclusions

Our results indicate that the US-AVP enhances safety by reducing radiation exposure and time to vascular access while maintaining a low rate of major complications compared to the X-ray-guided approach. Therefore, a US-guided approach may be regarded as optimal for venous access during CIED implantation; however, further large-scale real-world analyses are necessary to validate these preliminary findings.

## Supplementary Material

euae274_Supplementary_Data

## Data Availability

The experimental data used to support the findings of this study are available from the corresponding author upon reasonable request.

## References

[1] Burri H, Starck C, Auricchio A, Biffi M, Burri M, D’Avila A et al EHRA expert consensus statement and practical guide on optimal implantation technique for conventional pacemakers and implantable cardioverter-defibrillators: endorsed by the Heart Rhythm Society (HRS), the Asia Pacific Heart Rhythm Society (APHRS), and the Latin-American Heart Rhythm Society (LAHRS). Europace 2021;23:983–1008. 33878762 10.1093/europace/euaa367 PMC12378894

[2] Parsonnet V, Roelke M. The cephalic vein cutdown versus subclavian puncture for pacemaker/ICD lead implantation. Pacing Clin Electrophysiol 1999;22:695–7. 10353126 10.1111/j.1540-8159.1999.tb00531.x

[3] Benz AP, Vamos M, Erath JW, Hohnloser SH. Cephalic vs. subclavian lead implantation in cardiac implantable electronic devices: a systematic review and meta-analysis. Europace 2019;21:121–9. 30020452 10.1093/europace/euy165

[4] Atti V, Turagam MK, Garg J, Koerber S, Angirekula A, Gopinathannair R et al Subclavian and axillary vein access versus cephalic vein cutdown for cardiac implantable electronic device implantation: a meta-analysis. JACC Clin Electrophysiol 2020;6:661–71. 32553216 10.1016/j.jacep.2020.01.006

[5] Migliore F, Siciliano M, De Lazzari M, Ferretto S, Valle CD, Zorzi A et al Axillary vein puncture using fluoroscopic landmarks: a safe and effective approach for implantable cardioverter defibrillator leads. J Interv Card Electrophysiol 2015;43:263–7. 25956478 10.1007/s10840-015-0011-7

[6] Burri H, Sunthorn H, Dorsaz PA, Shah D. Prospective study of axillary vein puncture with or without contrast venography for pacemaker and defibrillator lead implantation. Pacing Clin Electrophysiol 2005;28:S280–3. 15683516 10.1111/j.1540-8159.2005.00039.x

[7] Charles P, Ditac G, Montoy M, Thenard T, Courand PY, Lantelme P et al Intra-pocket ultrasound-guided axillary vein puncture vs. cephalic vein cutdown for cardiac electronic device implantation: the ACCESS trial. Eur Heart J 2023;44:4847–58. 37832512 10.1093/eurheartj/ehad629 PMC10702459

[8] Stewart LA, Clarke M, Rovers M, Riley RD, Simmonds M, Stewart G et al Preferred Reporting Items for Systematic Review and Meta-Analyses of individual participant data: the PRISMA-IPD statement. JAMA 2015;313:1657–65. 25919529 10.1001/jama.2015.3656

[9] Jørgensen L, Paludan-Müller AS, Laursen DR, Savović J, Boutron I, Sterne JA et al Evaluation of the Cochrane tool for assessing risk of bias in randomized clinical trials: overview of published comments and analysis of user practice in Cochrane and non-Cochrane reviews. Syst Rev 2016;5:80. 27160280 10.1186/s13643-016-0259-8 PMC4862216

[10] Stang A . Critical evaluation of the Newcastle-Ottawa scale for the assessment of the quality of nonrandomized studies in meta-analyses. Eur J Epidemiol 2010;25:603–5. 20652370 10.1007/s10654-010-9491-z

[11] Vitali F, Malagù M, Bianchi N, De Raffele M, Manfrini M, Gibiino F et al Ultrasound-guided venous axillary access versus standard fluoroscopic technique for cardiac lead implantation: ZEROFLUOROAXI randomized trial. JACC Clin Electrophysiol 2024;10:554–65. 38243998 10.1016/j.jacep.2023.11.020

[12] Migliore F, Fais L, Vio R, De Lazzari M, Zorzi A, Bertaglia E et al Axillary vein access for permanent pacemaker and implantable cardioverter defibrillator implantation: fluoroscopy compared to ultrasound. Pacing Clin Electrophysiol 2020;43:566–72. 32394452 10.1111/pace.13940

[13] Courtney AM, Chandler JK, Anderson J, Shrestha A, Noheria A, Pimentel R et al UltraSound Axillary Vein Access (USAA): learning curve and randomized comparison to traditional venous access for cardiac device implantation. Pacing Clin Electrophysiol 2022;45:1364–71. 36270271 10.1111/pace.14611

[14] Tagliari AP, Kochi AN, Mastella B, Saadi RP, di Leoni Ferrari A, Saadi EK et al Axillary vein puncture guided by ultrasound vs cephalic vein dissection in pacemaker and defibrillator implant: a multicenter randomized clinical trial. Heart Rhythm 2020;17:1554–60. 32360827 10.1016/j.hrthm.2020.04.030

[15] Jiménez-Díaz J, Higuera-Sobrino F, Piqueras-Flores J, Pérez-Díaz P, González-Marín MA. Fluoroscopy-guided axillary vein access vs cephalic vein access in pacemaker and defibrillator implantation: randomized clinical trial of efficacy and safety. J Cardiovasc Electrophysiol 2019;30:1588–93. 31310038 10.1111/jce.14060

[16] Vurgun VK, Candemir B, Gerede DM, Goksuluk H, Altin AT, Akyurek O et al Extrathoracic subclavian-axillary vein location and morphological features over the first rib for pacemaker and defibrillator lead implantation. Pacing Clin Electrophysiol 2018;41:927–32.Epub ahead of print. 10.1111/pace.13396 29790579

[17] Yang F, Kulbak G. A new trick to a routine procedure: taking the fear out of the axillary vein stick using the 35° caudal view. Europace 2015;17:1157–60. 25969438 10.1093/europace/euv066

[18] Kirkfeldt RE, Johansen JB, Nohr EA, Moller M, Arnsbo P, Nielsen JC. Pneumothorax in 282 cardiac pacing: a population-based cohort study of 28,860 Danish patients. Europace 2012;14:1132–8. 22431443 10.1093/europace/eus054

[19] Malagù M, Trevisan F, Scalone A, Marcantoni L, Sammarco G, Bertini M. Frequency of “pocket” hematoma in patients receiving vitamin K antagonist and antiplatelet therapy at the time of pacemaker or cardioverter defibrillator implantation (from the POCKET Study). Am J Cardiol 2017;119:1036–40. 28153344 10.1016/j.amjcard.2016.12.012

[20] Sommer P, Sciacca V, Anselmino M, Tilz R, Bourier F, Lehrmann H et al Practical guidance to reduce radiation exposure in electrophysiology applying ultra low-dose protocols: a European Heart Rhythm Association review. Europace 2023;25:euad191. 37410906 10.1093/europace/euad191 PMC10365833

