# Supplementary material for: Ultrasound-guided vs. fluoro-guided axillary venous access for cardiac implantable electronic devices: a patient-based meta-analysis
Source: Europace. 2024 Oct 29;26(11):euae274. doi: 10.1093/europace/euae274 (PMC11579654; doi:10.1093/europace/euae274)
Supplement: euae274_Supplementary_Data [file euae274_supplementary_data.pdf]

Ultrasound-guided versus Fluoro-guided Axillary Venous Access for Cardiac Implantable Electronic Devices: A Patient-Based Meta-analysis

Ultrasound-guided versus Fluoro-guided Axillary Venous Access for Cardiac Implantable Electronic Devices: A Patient-Based Meta-analysis

Running Head: *Ultrasound vs. Fluoro-Guided Axillary Venous Access for pacemaker and defibrillator implantations*

Francesco Vitali, MD, PhD 1; Marco Zuin, MD, MSc 1; Paul Charles, MD 2; Javier Jiménez-Díaz, MD, PhD, 3; Seth H. Sheldon MD, 4; Ana Paula Tagliari, MD, MSc, PhD, 5,6; Federico Migliore, MD, PhD 7; Michele Malagù, MD, 1; Mathieu Montoy, MD 2; Felipe Higuera Sobrino, MD, 3; Alex M. Courtney MD, 2; Adriano Nunes Kochi, MD, MSc, 6; Samir Fareh, MD, 2; Matteo Bertini MD, PhD 1.

1. Cardiology Department, Sant'Anna University Hospital, University of Ferrara; Ferrara, Italy.
2. Hôpital de la Croix Rousse, Hospices Civils de Lyon, Lyon, France
3. Arrhythmia Unit, Cardiology Department, Hospital General Universitario of Ciudad Real, Ciudad Real, Spain.
4. Department of Cardiovascular Medicine, The University of Kansas Medical Center; Kansas City, KS; United States of America
5. Federal University of Rio Grande do Sul; Porto Alegre, Brazil.
6. Department of Cardiovascular Surgery, Hospital Mãe de Deus, Porto Alegre, Brazil.
7. Department of Cardiac, Thoracic and Vascular Sciences and Public Health, University of Padova, Padua, Italy

Supplementary Files

Supplementary Table 1. Complete search strategy. 3
Supplementary Table 2. Patient-level data collected for the study. 4
Supplementary Table 3. Techniques used to perform the axillary vein puncture in the revised investigations. 5
Supplementary Table 4. Inclusion and exclusion criteria in revised investigations. 6
Supplementary Table 5. ROB 2 Scale for risk of bias assessment.. 7
Supplementary table 6. New Castle Ottawa scale for quality assessment of prospective study included in the meta-analysis. 8
Supplementary Table7. Periprocedural complications in the reviewed studies. 9

Search Strategy
**(pacemaker OR implantable cardioverter defibrillator) AND axillary venous access AND (fluorguided OR ultrasound)**

Supplementary Table 1. Complete search strategy.

**Item**	**Variables**
Demographics	Age, Sex
Clinical and comorbidities	Body mass index, arterial hypertension, previous myocardial infarction, history of heart failure, atrial fibrillation, diabetes, chronic kidney disease and left ventricular ejection fraction
Drugs	Antiplatelets, anticoagulants
Device type	Pacemaker (mono, bicameral or biventricular) and ICD (mono, bicameral or biventricular), active fixation lead
Procedure	Puncture site (inside the pocket or on the skin), n° of axillary puncture, procedure duration time, fluoroscopy time, total procedure dose area product, time to vascular access, n° of attempts for vascular access, cross-over to different vascular access to imaging modality, axillary vein puncture.
In-hospital outcome	In-hospital mortality
Periprocedural complications	Pocket Infection
	Pocket Hematoma
	PNX
	Inadvertent arterial puncture

Supplementary Table 2. Patient-level data collected for the study. PNX: Pneumothorax.

	Vitali et al. [11]	Courtney et al. [13]	Jimenez et al. [15]	Charles et al. [7]	Migliore et al. [12]	Tagliari et al. [14]
**Fluoroscopic guided AVP - techniques used**						
	- Anteroposterior and Caudal views<br><br>- Venography if needed after 3 attempts<br><br>- AVP prior to skin incision or inside the pocket at operator choice	-	- Anteroposterior view<br><br>- Venography if needed after 5 attempts<br><br>- AVP prior to skin incision	-	- Anteroposterior view<br><br>- Venography if needed after 4 attempts<br><br>- AVP inside the pocket	-
**Ultrasound guided AVP - techniques used**						
	- AVP prior to skin incision or inside the pocket at operator choice	- AVP prior to skin incision	-	- AVP inside the pocket<br><br>- Small-footprint US transducer	- AVP prior to skin incision	- AVP prior to skin incision

Supplementary Table 3. Techniques used to perform the axillary vein puncture (AVP) in the revised investigations.

	Vitali et al. [11]	Courtney et al. [13]	Jimenez et al. [15]	Charles et al. [7]	Migliore et al. [12]	Tagliari et al. [14]
**Inclusion Criteria**						
	- age of >18 years<br><br>- indication for PM/ICD/CRT implantation or upgrading	- indication of PM/ICD implantation	- indication of PM/ICD implantation	- age of >18 years<br><br>- indication of PM/ICD implantation	- indication of PM/ICD implantation	- indication of PM/ICD implantation
**Exclusion Criteria**						
	- ongoing pregnancy<br><br>- inability to express informed Consent<br><br>- implant of a leadless PM or subcutaneous ICD<br><br>- surgery for battery depletion.	- history of previous implanted CIEDs<br><br>- lead repositioning or device replacement	- ipsilateral pacing lead<br><br>- ipsilateral lymphadenectomy<br><br>- upper limb venous thrombosis<br><br>- CRT indication	- history of previously implanted endocardial lead<br><br>- CRT indication<br><br>- impossible superior central venous access or need for vein preservation (e.g. for haemodialysis)	- history of previous implanted CIEDs<br><br>- CRT indication	- lead repositioning or device replacement<br><br>- upper limb venous thrombosis

Supplementary Table 4. Inclusion and exclusion criteria in revised investigations.

	D1	D2	D3	D4	D5	OVERALL
Courtney, A. M (2022) **[13]**	🟡	🟢	🟢	🟢	🟢	🟢
Tagliari, A. P (2020) **[14]**	🟡	🟢	🟢	🟢	🟢	🟢
Charles, P (2023) **[7]**	🟢	🟢	🟢	🟢	🟢	🟢
Vitali, F (2024) **[11]**	🟢	🟡	🟢	🟢	🟢	🟢
Jiménez-Díaz, J (2019) **[15]**	🟢	🟡	🟢	🟢	🟢	🟢

Supplementary Table 5. ROB 2 Scale for risk of bias assessment. **D1:** bias arising from the randomization process **D2:** bias due to the deviation from the intended interventions. **D3:** bias due to missing outcome data **D4:** bias in measurement of the outcome **D5:** bias in selection of the reported results.

	Selection				Comparability		Exposure			
**References**	**1**	**2**	**3**	**4**	**1**	**2**	**1**	**2**	**3**	**Total**
Migliore F. 2020 [12]	*	0	*	*	*	0	*	*	*	7/9

Supplementary table 6. New Castle Ottawa scale for quality assessment of prospective study included in the meta-analysis.

	Vitali et al. [11]	Courtney et al. [13]	Jimenez et al. [15]	Charles et al. [7]	Migliore et al. [12]	Tagliari et al. [14]
**Periprocedural Complications**						
PNX, n (%)	0	0	0	0	0	1 (2.3)
Hemothorax, n (%)	0	0	0	0	0	0
Pocket infection, n (%)	4 (1.5)	0	1 (0.8)	1 (1.0)	0	0
Pocket hematoma, n (%)	23 (8.5)	2 (2.0)	1 (0.8)	0	0	0
Acute lead dislodgment, n (%)	5 (1.9)	1 (1.0)	3 (2.5)	1 (1.0)	0	0
Inadvertent Axillary arterial puncture, n (%)	31 (11.5)	8 (8.0)	0	0	0	0
Intra-hospital mortality, n (%)	15 (5.6)	1 (1.0)	0	0	0	0

Supplementary Table7. Periprocedural complications in the reviewed studies. PNX: Pneumothorax.
